# Roxadustat Efficacy and Safety in Patients Receiving Peritoneal Dialysis: Pooled Analysis of Four Phase 3 Studies

**DOI:** 10.3390/jcm13226729

**Published:** 2024-11-08

**Authors:** Danilo Fliser, Sunil Bhandari, Alberto Ortiz, Vicki Santos, Najib Khalife, Alina Jiletcovici, Tadao Akizawa

**Affiliations:** 1 Saarland University Medical Center, 66424 Homburg, Germany; 2 Hull University Teaching Hospitals NHS Trust, Hull York Medical School, Hull HU3 2JZ, UK; sunil.bhandari@nhs.net; 3 UAM, IIS-Fundacion Jimenez Diaz, 28015 Madrid, Spain; aortiz@fjd.es; 4 Astellas Pharma, Inc., Northbrook, IL 60062, USA; vicki.santos@astellas.com (V.S.); alina.jiletcovici@astellas.com (A.J.); 5 Astellas Pharma Europe Ltd., Addlestone KT15 2NX, UK; najib.khalife@astellas.com; 6 Division of Nephrology, Department of Medicine, Showa University School of Medicine, Tokyo 142-8666, Japan; akizawa@med.showa-u.ac.jp

**Keywords:** anemia, chronic kidney disease, roxadustat, peritoneal dialysis

## Abstract

**Background/Objectives:** Roxadustat is an oral hypoxia-inducible factor prolyl hydroxylase inhibitor approved to treat anemia of chronic kidney disease (CKD). The efficacy and safety of roxadustat compared with parenteral erythropoiesis-stimulating agents (ESAs) were evaluated in patients with anemia of CKD receiving peritoneal dialysis (PD). **Methods:** This analysis pooled data from four phase 3, multicenter, randomized, open-label, active-comparator studies (PYRENEES, SIERRAS, HIMALAYAS, ROCKIES). The primary endpoints evaluated were hemoglobin change from baseline (CFB) to Weeks 28–36 without rescue therapy and hemoglobin CFB to Weeks 28–52 regardless of rescue therapy use. Safety data were reported. **Results:** This analysis included 422 patients (215 roxadustat, 207 ESA). Hemoglobin CFB to Weeks 28–36 without rescue therapy and hemoglobin CFB to Weeks 28–52 regardless of rescue therapy achieved non-inferiority for roxadustat vs. ESAs. The mean weekly dose of roxadustat was maintained over time (Weeks 1–4, 3.86 mg/kg/week; Weeks 101–104, 3.27 mg/kg/week), whereas the mean weekly ESA dose increased by 24% (Weeks 1–4, 115.70 IU/kg/week; Weeks 101–104, 143.40 IU/kg/week). Fewer patients treated with roxadustat received intravenous iron supplementation and rescue therapy, and patients treated with an ESA required blood transfusions sooner. Roxadustat-treated patients experienced a greater decrease in low-density lipoprotein cholesterol levels relative to baseline vs. ESA-treated patients. Treatment-emergent adverse events were similar in both treatment groups. Major adverse cardiovascular event (MACE), MACE plus unstable angina or congestive heart failure, and all-cause mortality hazard ratios were <1; the lower limit of the 95% CIs was <0.6, and the upper limit was >1.3. **Conclusions:** Roxadustat was non-inferior to ESAs in correcting and maintaining hemoglobin levels, with stable dosing and a comparable safety profile, in anemic patients receiving PD.

## 1. Introduction

Anemia, a common complication for patients with chronic kidney disease (CKD), is associated with decreased health-related quality of life (QoL), greater necessity for blood transfusions, and increased risk of cardiovascular (CV) events, hospitalization, and mortality [1,2]. Anemia occurs in approximately 15% of patients with CKD [3,4]; anemia prevalence increases with CKD severity [5,6]. Peritoneal dialysis (PD), a kidney replacement therapy modality, allows patients to manage their treatment at home and may result in clinical outcomes and QoL comparable to, or superior to, those achieved by patients on hemodialysis (HD) [7,8]. Anemia and iron deficiency are less prevalent in patients on PD compared with those on HD [9].

Among patients receiving PD, those with anemia have an increased risk of fatigue, hospitalization, and mortality [10]. Treatment of anemia of CKD can include iron supplementation and erythropoiesis-stimulating agents (ESAs) [11]. ESAs can improve hemoglobin levels but may increase the risk of adverse events, including mortality, stroke, and thromboembolic events, particularly in patients with cancer or CV disease [11]. Anemia of CKD occurs due to diminished erythropoietin production and/or disrupted iron homeostasis. ESA treatment does not address functional iron deficiency, while intravenous iron supplementation, due to its mode of administration, can further increase hepcidin levels [12].

Roxadustat, an oral medication, has a novel mechanism of action that addresses the multifactorial etiology of anemia of CKD. The transient inhibition of hypoxia-inducible factor prolyl hydroxylase (HIF-PH) induced by roxadustat mimics the body’s natural response to hypoxia [2], resulting in increased erythropoiesis, transferrin receptor expression, and iron uptake, thereby increasing hemoglobin levels [13]. Roxadustat is approved in multiple countries to treat anemia of non–dialysis-dependent and dialysis-dependent (DD) CKD.

PD is the starting modality of kidney replacement therapy in 11% of patients with kidney failure [14], and the PD patient population is growing [8]. As an oral medication, roxadustat may allow PD patients to treat their anemia at home rather than in a dialysis facility or be trained to administer a parenteral ESA. A previous pooled analysis examined the efficacy and safety of roxadustat in a large, global population of patients with anemia of DD CKD, which included patients receiving PD or HD [15]. The objective of the current pooled subgroup analysis was to examine the efficacy and safety of roxadustat compared with ESAs in a global population of patients with anemia of CKD restricted to only those receiving PD.

## 2. Materials and Methods

### 2.1. Component Studies and Pooling Methodology

**Figure 1:**
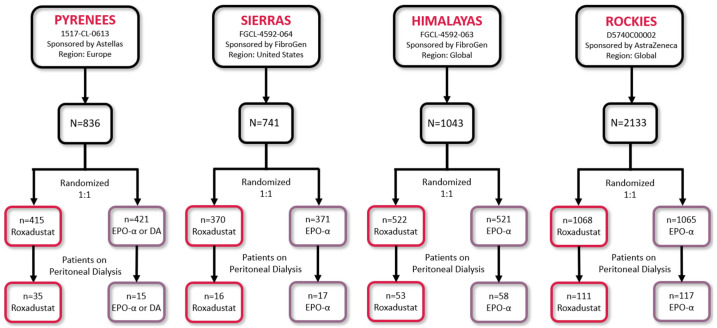
Studies included in the pooled analysis. DA, darbepoetin alfa; EPO-α, epoetin alfa.

Four phase 3, multicenter, randomized, open-label, active-comparator studies (PYRENEES [NCT02278341]; SIERRAS [NCT02273726]; HIMALAYAS [NCT02052310]; and ROCKIES [NCT02174731]) were included in this pooled analysis (Figure 1) [16,17,18,19].

Prior to enrollment, all patients provided informed written consent. The studies were conducted in accordance with the ethical principles of the Declaration of Helsinki and the International Council for Harmonisation, guidelines for Good Clinical Practice, and were reviewed and approved by relevant institutional review boards and/or ethics committees. Individual study details are available at https://clinicaltrials.gov/ct2/show/NCT02278341; https://clinicaltrials.gov/ct2/show/NCT02273726; https://clinicaltrials.gov/ct2/show/NCT02052310; https://clinicaltrials.gov/ct2/show/NCT02174731 (all accessed on 5 September 2024); and their associated publications [16,17,18,19].

### 2.2. Participants

Eligible patients from the Global ALPINE Roxadustat Clinical Program were adults (≥18 years) with anemia of CKD on PD and were iron-replete (ferritin ≥ 100 ng/mL and transferrin saturation [TSAT] ≥ 20%). Participants were excluded if they had a recent red blood cell (RBC) transfusion (≤4 weeks before randomization [HIMALAYAS; SIERRAS]; ≤8 weeks before randomization [PYRENEES]; or anytime during the screening period [ROCKIES]), prior treatment with roxadustat or another HIF-PH inhibitor, or active/chronic gastrointestinal bleeding, or if they anticipated elective surgery with blood loss expected.

Patients were randomized (1:1) to receive an ESA (epoetin alfa [PYRENEES, SIERRAS, HIMALAYAS, ROCKIES] or darbepoetin alfa [PYRENEES]) or oral roxadustat. The hemoglobin threshold, prior to randomization, was 9.0–12.0 g/dL.

### 2.3. Interventions and Rescue Therapy [20]

Initial drug dosing was based on body weight for ESA-untreated patients and average weekly ESA dose before randomization for ESA-pretreated patients. Roxadustat dose was adjusted every 4 weeks per prespecified rules to maintain hemoglobin between 10 and 12 g/dL. ESA doses followed local labeling and guidelines.

Rescue therapy was a blood product transfusion and ESAs (roxadustat treatment arm only). Additional information regarding rescue therapy and iron administration protocols is in the Supplementary Methods.

### 2.4. Efficacy Endpoints

The primary endpoints in this post hoc analysis were hemoglobin change from baseline (CFB) to Weeks 28–36 without rescue therapy and to Weeks 28–52 regardless of rescue therapy use. Secondary endpoints included hemoglobin CFB to Weeks 18–24, regardless of rescue therapy, for patients with baseline high-sensitivity C-reactive protein (hsCRP) above the upper limit of normal (ULN); CFB in low-density lipoprotein cholesterol (LDL-C) to Weeks 12–28; time to first RBC/blood transfusion during treatment; proportion of patients receiving intravenous iron supplementation during treatment; and proportion of patients using rescue therapy during treatment. Exploratory outcomes included mean hemoglobin levels up to Week 104, mean weekly total roxadustat/ESA dose every 4 weeks up to Week 104, and iron parameters CFB to Week 24 (serum hepcidin) or Week 36 (serum iron, TSAT, and ferritin).

### 2.5. Adverse Events

Overall treatment-emergent adverse events (TEAEs) from the first study drug administration up to 28 days after the last dose (on-treatment period plus 28 days [OT-28]) and TEAEs with an incidence of ≥5% at OT-28 were reported for the safety analysis set (those who received ≥1 dose of study drug).

### 2.6. Safety Endpoints

Cardiovascular safety endpoints were time to first major adverse cardiovascular event (MACE; a composite of all-cause mortality [ACM], myocardial infarction, or stroke) and MACE plus (MACE+; a composite of MACE plus unstable angina or congestive heart failure requiring hospitalization up to 7 days after the last dose). Definitions for CV endpoints were based on the 2014 American College of Cardiology/American Heart Association Key Data Elements and Definitions for Cardiovascular Endpoint Events in Clinical Trials [21]. All CV safety endpoints were adjudicated by a central Independent Event Review Committee, and members were blinded to treatment assignment.

### 2.7. Statistical Analysis

A chi-squared test or Wilcoxon rank-sum test was used to compare the demographics and baseline clinical characteristics of the treatment groups. Mean weekly total doses of roxadustat or ESA are presented with descriptive statistics.

Statistical methods used to analyze the data from patients undergoing peritoneal dialysis were consistent with those previously reported [15]. The methods defined in the a priori-developed statistical analysis plan for the entire dialysis population were followed when analyzing the mean hemoglobin change from baseline for the subset of patients with peritoneal dialysis. The statistical analysis plan did not include a statement to analyze the peritoneal dialysis subset.

The mean hemoglobin CFB to Weeks 28–36, to Weeks 28–52, and to Weeks 18–24 were compared between the two treatment groups using the least squares (LS) mean values and the LS mean difference (LSMD) with 95% confidence intervals (CIs). The margin for the lower limit of the CI for the difference between roxadustat and ESA used to establish non-inferiority was defined as −0.75 g/dL.

The mean CFB in LDL-C, serum hepcidin, serum iron, TSAT, and ferritin were compared using the LSMD between the treatment groups and the 95% CI. ANCOVA models for each analysis are provided in the Supplementary Methods and Table S1.

The exact method of Clopper–Pearson was used to determine the proportion of patients who required intravenous iron supplementation and the 95% CI for the difference. The treatment groups were compared using a chi-square test.

A stratified Cox proportional hazards model was used to compare the treatment groups for time to first RBC blood transfusion, time to ESA rescue, and the time to either RBC blood transfusion or ESA rescue. Covariates studied in the model were baseline hemoglobin (<10 g/dL vs. ≥10 g/dL) and a history of CV/cerebrovascular/thromboembolic disease (yes vs. no). Incidence rates (IRs) per 100 patient exposure years (PEYs) are provided for patients who had an RBC transfusion, used ESA as rescue, and those who had either of these. Total PEYs were calculated as follows:

([last dose date − first dose date] + 1)/365.25

The HR and the 95% CI are presented and were derived from a Cox proportional hazards model adjusting for treatment and stratified by study, baseline hemoglobin (<10 g/dL vs. ≥10 g/dL), and history of CV/cerebrovascular/thromboembolic disease (yes vs. no).

The closed testing procedure to control the family-wise Type I error rate that was applied to all patients receiving dialysis was not applied in these post hoc analyses of patients receiving PD.

TEAEs were presented with counts, percentages, and IRs per PEY. MACE, MACE+, and ACM were presented with counts, percentages, and follow-up adjusted IRs. Patient years per follow-up adjusted incidence rates were calculated as ([first event occurrence or censor date − first dose date] + 1)/365.25. These analysis methods and the pooling process to determine the HR have been previously described [15]. The analysis period for MACE, MACE+, and ACM was the on-treatment period defined in the European Medicines Agency guidelines. On-treatment includes an additional 7 days after the last dose of the study drug [22]. All analyses were performed with SAS^®^ Version 9.3 or higher.

## 3. Results

### 3.1. Patients

Of the 422 patients in this analysis, 215 received roxadustat and 207 received an ESA (epoetin alfa or darbepoetin alfa; Figure 1). Demographic and baseline characteristics were similar for the two treatment groups, but there was a significantly higher proportion of patients receiving roxadustat who were white (Table 1). Most patients were iron-replete at baseline (87.0%, roxadustat group; 91.3%, ESA group). Mean baseline hemoglobin (9.8 g/dL, roxadustat; 9.7 g/dL, ESAs) and LDL-C levels (114.6 mg/dL, roxadustat; 108.6 mg/dL, ESAs) were similar for both treatment groups. Hepcidin levels were elevated in both groups and statistically greater at baseline in those who were randomized to the ESA group. There were 79 roxadustat-treated patients (36.7%) and 67 ESA-treated patients (32.4%) with baseline hsCRP levels > ULN. The mean duration of treatment exposure was similar for roxadustat (81.6 weeks) and ESAs (82.8 weeks; Table S2).

### 3.2. Efficacy Endpoints

Hemoglobin CFB to Weeks 28–36 without rescue therapy following roxadustat treatment (LS mean: 1.38 g/dL; 95% CI: 1.21, 1.56) was non-inferior to the change following ESA treatment (LS mean: 0.97 g/dL; 95% CI: 0.78, 1.16; [change for roxadustat] − [change for ESA] LSMD: 0.41 g/dL; 95% CI: 0.16, 0.67). Hemoglobin CFB to Weeks 28–52 regardless of rescue therapy for roxadustat (LS mean: 1.31 g/dL; 95% CI: 1.15, 1.47) was non-inferior to the change for ESAs (LS mean: 1.00 g/dL; 95% CI: 0.82, 1.17; [change for roxadustat] − [change for ESA] LSMD: 0.32 g/dL; 95% CI: 0.08, 0.55; Table 2).

For patients with chronic inflammation (defined as a baseline hsCRP level > ULN), the LS mean (95% CI) hemoglobin CFB to Weeks 18–24, regardless of rescue therapy, was 1.42 g/dL (1.14, 1.70) for roxadustat and 1.08 g/dL (0.74, 1.42) for ESAs **(**Table 2). The LSMD (95% CI) was 0.34 g/dL (−0.08, 0.77), so roxadustat was non-inferior, with a lower confidence limit > −0.75, for chronically inflamed patients.

**Figure 2:**
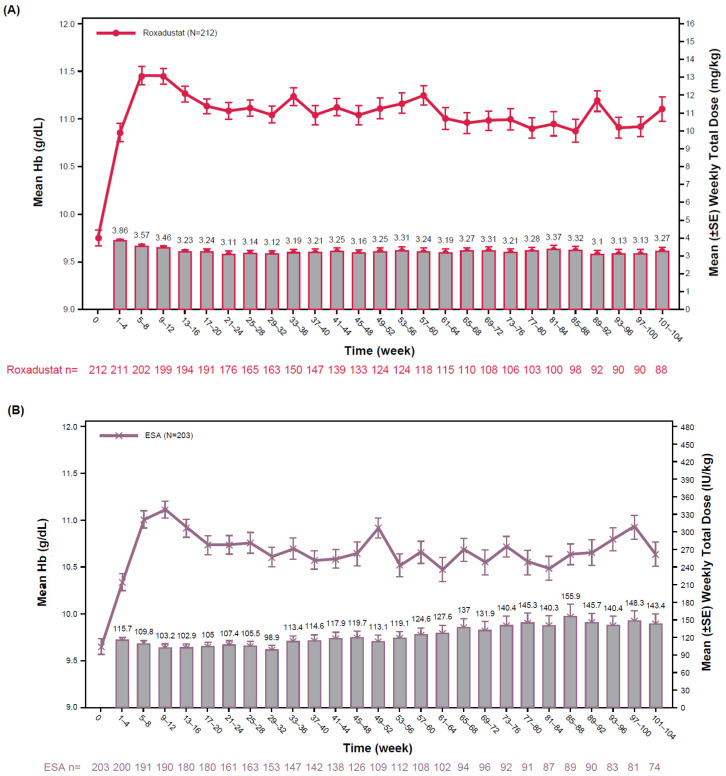
Mean Hb levels (g/dL, lines) and mean weekly total dose (columns) of roxadustat (**A**) or ESA (**B**) from baseline to Week 104. ESA, erythropoiesis-stimulating agent; Hb, hemoglobin; SE, standard error.

For patients with chronic inflammation, treatment with either roxadustat or ESA increased hemoglobin; ESA doses were increased by 75.0% (Weeks 1–4, 123.40 IU/kg/week; Weeks 101–104, 216.00 IU/kg/week) and roxadustat doses remained stable (Figure S1). Within 8 weeks, both treatment groups achieved a substantial increase in mean hemoglobin levels. The mean weekly roxadustat dose gradually decreased during the initial 16 weeks and was relatively maintained thereafter up to 104 weeks (Weeks 1–4, 3.86 mg/kg/week; Weeks 101–104, 3.27 mg/kg/week). The mean weekly ESA dose gradually increased beginning in the Week 33–36 interval; the mean weekly ESA dose increased by 23.9% (Weeks 1–4, 115.70 IU/kg/week; Weeks 101–104, 143.40 IU/kg/week; Figure 2).

**Figure 3:**
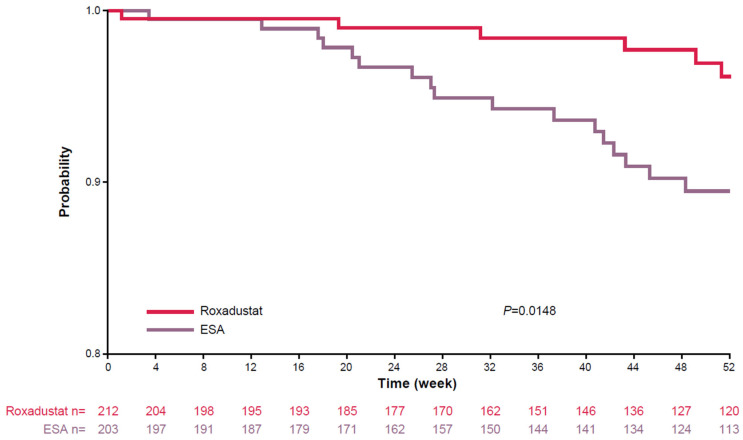
Time to rescue therapy with RBC transfusion for patients treated with roxadustat or ESA from baseline to Week 52. *p* value determined by log-rank test. ESA, erythropoiesis-stimulating agent; RBC, red blood cell.

Fewer roxadustat-treated patients (n = 88, 41.5%) required intravenous iron supplementation during treatment compared with ESA-treated patients (n = 166, 81.8%; *p* < 0.0001). Roxadustat-treated patients were less likely to need an RBC transfusion (n = 17, 8.0%) compared with ESA-treated patients (n = 28, 13.8%), and ESA-treated patients required RBCs sooner than roxadustat-treated patients (HR: 0.49; 95% CI: 0.25, 0.93; *p* = 0.030; Table 2, Figure 3). Rescue therapy (RBC therapy in the ESA treatment group, RBC or ESA therapy in the roxadustat treatment group) was required in 22 roxadustat-treated patients (10.4%) and in 28 ESA-treated patients (13.8%; HR: 0.70; 95% CI: 0.39, 1.27; *p* = 0.244; Table 2). Among patients with chronic inflammation, the percentage of roxadustat-treated patients (14.1%) who required rescue therapy was similar to the percentage of ESA-treated patients (9.1%; HR: 1.43; 95% CI: 0.45, 4.51; *p* = 0.546; Table 2).

There was a decrease relative to baseline in LDL-C to Weeks 12–28 for the roxadustat-treated patients (LS mean: −7.82 mg/dL; 95% CI: −13.23, −2.40) compared with the ESA-treated patients (LS mean: 1.66 mg/dL; 95% CI: −4.48, 7.80; [CFB for roxadustat] − [CFB for ESA] LSMD: −9.47; 95% CI: −17.41, −1.54; *p* = 0.019; Table 2).

Although the changes in iron parameters between the treatment arms did not reach statistical significance in this exploratory analysis, the results were consistent with previous findings. The LS mean decrease from baseline (SEM) to Week 24 in serum hepcidin levels was −35.0 (20.2) for roxadustat and −3.0 (22.7) for ESAs (*p* = 0.1537). The LS mean decreases from baseline to Week 36 in ferritin, and TSAT levels were numerically lower in the roxadustat treatment group compared with the LS mean decreases in the ESA group. Serum iron levels were relatively stable in the roxadustat treatment group and numerically decreased in the ESA treatment group (Table 3).

### 3.3. Safety Endpoints

Overall TEAEs (IRs: 56.5/100 PEY vs. 54.4/100 PEY) and serious TEAEs (IRs: 37.2/100 PEY vs. 35.8/100 PEY) were similar in the roxadustat and ESA groups, respectively. The IR for TEAEs leading to study drug discontinuation was 5.7/100 PEY for roxadustat and 3.4/100 PEY for ESA. The IR for TEAEs leading to death was 8.0/100 PEY in both treatment groups (Table 4). The most common TEAEs (≥5% of patients [OT-28]) were peritonitis, hypertension, hypotension, diarrhea, and nausea (Table S3). IRs for MACE, MACE+, and ACM were 8.0, 8.9, and 5.7, respectively, for roxadustat-treated patients and were 9.0, 11.7, and 6.2, respectively, for ESA-treated patients, with HRs of 0.96 (MACE), 0.89 (MACE+), and 0.96 (ACM). However, the 95% CI included 1 for MACE, MACE+, and ACM, indicating no statistical difference between the two groups for any of these parameters. In this small patient population, the upper limits of the 95% CIs all exceeded 1.3 (Table 5). The individual components for MACE, MACE+, and mortality are reported in Table 6 and Table 7. IRs/100 PEY for myocardial infarction, stroke, and hospitalization for congestive heart failure were 1.8, 1.2, and 1.2, respectively, for the roxadustat treatment group and were 2.5, 1.6, and 3.2, respectively, for the ESA treatment group in the PD population. IRs for CV-related and non-CV-related mortality were 2.4 and 2.1, respectively, with roxadustat treatment and 3.4 and 2.5, respectively, with ESA treatment.

## 4. Discussion

The results of this pooled analysis indicate that roxadustat is an alternative to the current standard of care for patients with anemia of CKD receiving PD. Roxadustat demonstrated non-inferiority compared with ESAs for hemoglobin CFB to Weeks 28–36 for patients without rescue therapy and hemoglobin CFB to Weeks 28–52 regardless of rescue therapy with target hemoglobin levels of 10–12 g/dL. The mean weekly dose of roxadustat was maintained over time up to 104 weeks (Weeks 1–4, 3.86 mg/kg/week; Weeks 101–104, 3.27 mg/kg/week), while the mean weekly dose of ESAs increased beginning at Weeks 41–44 (Weeks 1–4, 115.70 IU/kg/week; Weeks 101–104, 143.40 IU/kg/week). This difference in dose requirements was more pronounced in patients with high baseline hsCRP, who are prone to needing escalating ESA doses, which is a risk factor for adverse clinical outcomes.

In a recent pooled analysis including the largest global population of patients receiving HD or PD from the same four phase 3 trials as in the current study, roxadustat demonstrated non-inferiority for hemoglobin CFB to Weeks 28–36 compared with ESAs [15]. Patients with elevated hsCRP levels treated with an ESA required increasing doses to maintain hemoglobin levels over time compared with roxadustat-treated patients, in whom the dose was stable. While multiple potential etiologies exist for elevated CRP, a recent study in patients with anemia of CKD on HD indicated there may be a causal relationship between elevated CRP levels and ESA hyporesponsiveness [23]. Roxadustat may be more effective than ESAs to treat anemia of CKD in patients with ESA hyporesponsiveness [24]. The findings reported here are consistent with these publications.

Studies conducted on patients receiving PD in Japan [25] and in China [26] found that roxadustat increased and then maintained target hemoglobin levels with an acceptable safety profile [25]. These findings are also supported by real-world evidence studies [27,28,29,30]. In a prior analysis, the change from baseline to Weeks 28–36 in hemoglobin levels was non-inferior with roxadustat treatment compared with active control, regardless of dialysis modality [15].

Significantly fewer roxadustat-treated patients received intravenous iron supplementation or an RBC transfusion compared with ESA-treated patients. ESA-treated patients required RBC/blood transfusion sooner than roxadustat-treated patients did. Patients on PD are often candidates for kidney transplantation [31,32]. A prior report determined that 20% of patients who received a blood transfusion developed a biologically relevant increase in allosensitization compared with <2% of matched controls [33]. Similarly, previous studies have found that blood transfusions may result in clinically meaningful increases in human leukocyte antigen antibody strength and breadth, leading to sensitization and reducing the likelihood of receiving a transplant due to donors becoming incompatible [34], as well as accelerating allograft loss following a transplant [34,35]. Roxadustat treatment likely results in less supplemental intravenous iron usage and RBC transfusions due to increased erythropoiesis and improved iron availability and utilization [36,37]. The requirement for fewer RBC transfusions with roxadustat may benefit patients who require a kidney transplant and could result in better transplant-associated outcomes for patients with anemia of CKD.

Consistent with previous studies in patients with DD CKD, TEAE outcomes were generally similar for roxadustat- and ESA-treated patients [15,38]. Patients receiving PD may have a lower risk of mortality compared to patients receiving HD, at least in the initial 2–3 years of dialysis [14]. In the present study, the IRs for mortality related to MACE and MACE+ were similar between the roxadustat- and ESA-treated groups. Additionally, the IRs for MACE, MACE+, and ACM were numerically lower for roxadustat-treated patients compared with ESA-treated patients. These findings are similar to the results from Barratt and colleagues, who assessed the efficacy and safety of roxadustat compared with ESAs using combined HD and PD patient data from the same four phase 3 trials as in the current study [15]. Additionally, the IRs for infections, including peritonitis, were similar for the roxadustat and ESA treatment groups. Although the mechanism of action of HIF-PH inhibitors could theoretically lead to tumor growth, most clinical trials have not reported an increased risk of malignancies following HIF-PH inhibitor treatment [39]. It has been suggested that treatment with HIF-PH inhibitors may lead to or exacerbate retinopathy. However, a dedicated analysis of retinal photographs assessed by two independent blinded assessors did not show any difference in retinal hemorrhage between roxadustat and darbepoetin [40]; additionally, this has not been shown in clinical studies of roxadustat [41,42]. Hypothyroidism is prevalent in patients with advanced CKD [43], and roxadustat treatment may be associated with central hypothyroidism [44,45,46]. Monitoring of thyroid function is recommended for patients treated with HIF-PH inhibitors, including roxadustat. This pooled analysis was not designed to statistically test for significant differences in IRs of TEAEs in patients treated with roxadustat versus ESAs and was not designed to have sufficient power to test for non-inferiority for MACE, MACE+, and ACM in this limited set of patients with PD.

There was a significant decrease from baseline to Weeks 12–28 in LDL-C levels for patients treated with roxadustat compared with ESA. This finding is consistent with a prior study that reported that treatment with roxadustat in patients with anemia of DD CKD decreased LDL-C levels independent of statins and sevelamer [47]. Further study into the potential effects of decreased LDL-C levels on CV outcomes is warranted.

The current study had several strengths and limitations. This study included a large, global PD patient population, and PD patients are usually less studied than patients receiving HD. While some variables related to the type and effectiveness of dialysis therapy were unable to be evaluated, the overall number of participants compares favorably with other clinical trials in patients receiving PD [48,49,50]. The four studies included in this analysis evaluated similar efficacy and safety outcomes, thereby improving the confidence in the results presented here. This study did not observe any safety signals that would indicate patients receiving PD would be at a greater risk for complications with roxadustat treatment compared with ESAs. Roxadustat showed similar efficacy for patients receiving PD compared with the overall DD patient population. The four studies included in this analysis were open-label, which introduces a potential for bias when reporting the results for patients treated with roxadustat versus the standard of care (ESAs). This may explain, in part, why patients more frequently discontinued roxadustat compared with ESAs, as most of the TEAEs appear to be related to tolerability rather than safety concerns. Every 4 weeks, patients treated with roxadustat were permitted a dose adjustment to maintain hemoglobin levels in the range of 10–12 g/dL. Dosing in the ESA treatment group followed local labeling, so dose adjustments may have differed in frequency compared with the roxadustat treatment group.

A recent publication from the European Renal Best Practice Board of the European Renal Association suggests that HIF-PH inhibitors, including roxadustat, have several potential advantages compared with ESA therapy in patients with DD CKD receiving PD [51]. Overall, this pooled analysis demonstrates that roxadustat is non-inferior to ESAs (epoetin alfa and darbepoetin alfa) in correcting and maintaining hemoglobin levels, with a comparable safety profile, by improving the oxygen sensing pathway in patients with anemia of CKD receiving PD.

## Figures and Tables

**Table 1:** Pooled sample demographics and baseline characteristics.

Parameter	Roxadustat N = 215	ESA N = 207	*p* Value
Sex (male), n (%)	118 (54.9)	108 (52.2)	0.5769 ^a^
Age ^b^ (y), mean (SD)	51.0 (15.1)	52.7 (15.0)	0.2091 ^c^
Race, n (%)			0.0342 ^a^
White	147 (68.4)	110 (53.1)	
Black or African American	20 (9.3)	29 (14.0)	
Asian	29 (13.5)	41 (19.8)	
American Indian or Alaska Native	11 (5.1)	17 (8.2)	
Native Hawaiian or other Pacific Islander	0	0	
Other	8 (3.7)	10 (4.8)	
Body weight (kg), mean (SD)	75.1 (19.4)	74.8 (19.4)	0.9284 ^c^
CKD etiology, n (%) ^d^			0.7159 ^a^
Diabetic nephropathy	64 (29.8)	70 (33.8)	
Hypertensive nephropathy	51 (23.7)	51 (24.6)	
Other	116 (54.0)	104 (50.2)	
Cardiac, cerebrovascular, or TE disease, n (%)	73 (34.0)	71 (34.3)	0.9403 ^a^
Baseline Hb (g/dL), mean (SD)	9.8 (1.2)	9.7 (1.2)	0.2531 ^c^
Iron replete (TSAT ≥ 20% and ferritin ≥ 100 ng/mL) at baseline, n (%)	187 (87.0)	189 (91.3)	0.1539 ^a^
Baseline hsCRP (mg/L), mean (SD)	12.1 (23.6)	10.7 (36.3)	0.1500 ^c^
Baseline hsCRP, n (%)			0.3932 ^a^
≤ULN	107 (49.8)	109 (52.7)	
>ULN	79 (36.7)	67 (32.4)	
Missing	29 (13.5)	31 (15.0)	
Baseline LDL-C (mg/dL), mean (SD)	114.6 (47.4)	108.6 (47.9)	0.1462 ^c^
Baseline serum hepcidin (µg/L), mean (SD)	192.7 (152.3)	238.6 (163.6)	0.0039 ^c^
Baseline serum iron (µmol/L), mean (SD)	13.6 (6.3)	14.1 (7.1)	0.4425 ^c^
Baseline ferritin (ng/mL), mean (SD)	490.6 (413.8)	529.8 (391.0)	0.1500 ^c^
Dialysis vintage ^e^, n (%)			0.3684 ^a^
≤4 months	79 (36.7)	93 (44.9)	
>4 months	136 (63.3)	114 (55.1)	

CKD, chronic kidney disease; ESA, erythropoiesis-stimulating agent; Hb, hemoglobin; hsCRP, high-sensitivity C-reactive protein; LDL-C, low-density lipoprotein cholesterol; SD, standard deviation; TE, thromboembolic; TSAT, transferrin saturation; ULN, upper limit of normal. ^a^ *p* value determined by chi-squared test. ^b^ Age at completion of the informed consent or first dose date. ^c^ *p* value determined by Wilcoxon rank-sum test as data were not normally distributed. ^d^ Subjects may have more than one CKD etiology. ^e^ Relative to randomization. The category ≤ 4 months includes all participants in the HIMALAYAS study and patients in the PYRENEES, SIERRAS, and ROCKES studies that meet the criterion.

**Table 2:** Efficacy endpoints.

Endpoint/Parameter	Roxadustat	ESA
CFB in Hb (g/dL) to Weeks 28–36, without rescue therapy		
N	169	165
Baseline ^a^, mean (SD)	9.75 (1.22)	9.65 (1.24)
CFB, mean (SD)	1.37 (1.44)	0.98 (1.59)
LS mean (95% CI)	1.38 (1.21, 1.56)	0.97 (0.78, 1.16)
LSMD ^b^ (95% CI)	0.41 (0.16, 0.67)	
Non-inferiority margin (−0.75) for lower limit of LSMD 95% CI	−0.75 < 0.16, non-inferiority is met	
CFB in Hb (g/dL) to Weeks 28–52, regardless of rescue therapy		
N	215	207
Baseline ^a^, mean (SD)	9.76 (1.22)	9.65 (1.23)
CFB, mean (SD)	1.27 (1.38)	1.02 (1.49)
LS mean (95% CI)	1.31 (1.15, 1.47)	1.00 (0.82, 1.17)
LSMD ^c^ (95% CI)	0.32 (0.08, 0.55)	
Non-inferiority margin (−0.75) for lower limit of LSMD 95% CI	−0.75 < 0.08, non-inferiority is met	
CFB in Hb (g/dL) to Weeks 18–24, regardless of rescue therapy, for patients with baseline hsCRP >ULN		
N	78	66
Baseline ^a^, mean (SD)	9.84 (1.15)	9.56 (1.32)
CFB, mean (SD)	1.34 (1.31)	1.21 (1.50)
LS mean (95% CI)	1.42 (1.14, 1.70)	1.08 (0.74, 1.42)
LSMD ^c^ (95% CI)	0.34 (−0.08, 0.77)	
Non-inferiority margin (−0.75) for lower limit of LSMD 95% CI	−0.75 < −0.08, non-inferiority is met	
CFB in LDL-C (mg/dL) to Weeks 12–28		
N	199	194
Baseline ^d^, mean (SD)	115.03 (47.36)	108.52 (47.98)
CFB, mean (SD)	−11.76 (33.23)	−3.92 (32.07)
LS mean (95% CI)	−7.82 (−13.23, −2.40)	1.66 (−4.48, 7.80)
LSMD ^e^ (95% CI)	−9.47 (−17.41, −1.54)	
*p* value	0.019	
Proportion of patients receiving intravenous iron supplementation during treatment		
N	212	203
Patients with events, n (%)	88 (41.5)	166 (81.8)
95% CI ^f^	9.3, 13.9	19.4, 25.6
Treatment effect, response rate difference (95% CI) ^f^	−11.0 (−14.72, −7.22)	
*p* value ^g^	<0.0001	
Time to first RBC/blood transfusion during treatment		
N	212	203
Patients with events ^h^, n (%)	17 (8.0)	28 (13.8)
Total PEY ^i^	336.1	323.7
IR/100 PEY	5.1	8.7
Treatment effect, HR (95% CI) ^j^	0.49 (0.25, 0.93)	
*p* value ^j^	0.030	
Time to first ESA use during treatment		
N	212	203
Patients with events ^h^, n (%)	5 (2.4)	1 (0.5)
Total PEY ^i^	336.1	323.7
IR/100 PEY	1.5	0.3
Treatment effect, HR (95% CI) ^j^	6.96 (0.80, 60.58)	
*p* value ^j^	0.079	
Time to first rescue therapy (RBC/blood transfusion or ESA use) during treatment		
N	212	203
Patients with events ^h^	22 (10.4)	28 (13.8)
Total PEY ^i^	336.1	323.7
IR/100 PEY	6.5	8.7
Treatment effect, HR (95% CI) ^j^	0.70 (0.39, 1.27)	
*p* value ^j^	0.244	
Time to first RBC/blood transfusion during treatment for patients with hsCRP > ULN		
N	78	66
Patients with events ^h^	7 (9.0)	6 (9.1)
Total PEY ^i^	122.6	100.9
IR/100 PEY	5.7	5.9
Treatment effect, HR (95% CI) ^j^	0.74 (0.19, 2.89)	
*p* value ^j^	0.667	
Time to first ESA use during treatment for patients with hsCRP > ULN		
N	78	66
Patients with events ^h^	4 (5.1)	0 (0.0)
Total PEY ^i^	122.6	100.9
IR/100 PEY	3.3	0.0
Treatment effect, HR (95% CI) ^j^	NA (NA, NA) ^k^	
*p* value	NA ^k^	
Time to first rescue therapy (RBC/blood transfusion or ESA use) during treatment for patients with hsCRP > ULN		
N	78	66
Patients with events ^h^	11 (14.1)	6 (9.1)
Total PEY ^i^	122.6	100.9
IR/100 PEY	9.0	5.9
Treatment effect, HR (95% CI) ^j^	1.43 (0.45, 4.51)	
*p* value ^j^	0.546	

PEY for each patient = ([last dose date − first dose date] + 1)/365.25. ANCOVA, analysis of covariance; CFB, change from baseline; CI, confidence interval; ESA, erythropoiesis-stimulating agent; Hb, hemoglobin; HR, hazard ratio; hsCRP, high-sensitivity C-reactive protein; IR, incidence rate; IR/100 PEY = 100 × number of patients with events/PEY; OT-28, on-treatment period plus 28 days; LDL-C, low-density lipoprotein cholesterol; LS, least squares; LSMD, least squares mean difference; NA, not applicable; PEY, patient exposure years; RBC, red blood cell; SD, standard deviation; ULN, upper limit of normal. ^a^ Baseline Hb is defined as the mean of up to four last central lab values prior to the first dose of study treatment. ^b^ Treatment comparison was made using a mixed model of repeated measures with baseline Hb as a covariate, and study, treatment, visit, visit-by-treatment interaction, study-by-treatment interaction, and history of cardiovascular/cerebrovascular/thromboembolic disease (yes vs. no) as fixed effects. ^c^ Treatment comparison was made using the multiple imputation strategy by combining the results of an ANCOVA model with baseline Hb as covariate and study, treatment, study-by-treatment interaction, and history of cardiovascular/cerebrovascular/thromboembolic disease (yes vs. no) as fixed effects. ^d^ Baseline is defined as the last available value prior to the first dose of study treatment. ^e^ Treatment comparison was made using an ANCOVA model with baseline Hb, baseline LDL-C as covariates and study, treatment, study-by-treatment interaction, and history of cardiovascular/cerebrovascular/thromboembolic disease (yes vs. no) as fixed effects. ^f^ 95% CI for responder rate for roxadustat and ESA was based on the exact method of Clopper-Pearson. ^g^ *p* value was determined using chi-square testing. ^h^ Subjects with no event were censored at the date of minimum (last dose date, last visit date, death date). ^i^ Total PEY was calculated as ([last dose date − first dose date] + 1)/365.25. ^j^ From a stratified Cox proportional hazards model adjusting for treatment stratified by study, baseline hemoglobin (<10 g/dL vs. ≥10 g/dL), and history of cardiovascular/cerebrovascular/thromboembolic disease (yes vs. no). ^k^ Not applicable because proportional hazards assumption was violated.

**Table 3:** Iron parameters (serum hepcidin, serum iron, TSAT, ferritin).

Parameter	Roxadustat	ESA
CFB in serum hepcidin (µg/L) to Week 24		
N	164	157
Baseline, mean (SD)	192.69 (152.32)	239.07 (164.01)
Week 24 n	152	136
CFB, mean (SD)	−47.52 (139.59)	−18.25 (137.76)
LS mean (95% CI)	−35.00 (−74.87, 4.86)	−2.99 (−47.85, 41.87)
LSMD ^a^ (95% CI)	−32.01 (−76.11, 12.08)	
*p* value	0.154	
CFB in serum iron (µg/dL) to Week 36		
N	212	203
Baseline, mean (SD)	75.51 (34.77)	78.39 (39.66)
Week 36 n	153	151
CFB, mean (SD)	6.42 (48.96)	−9.89 (48.00)
LS mean (95% CI)	−1.19 (−14.07, 11.70)	−8.47 (−22.59, 5.65)
LSMD ^b^ (95% CI)	7.28 (−6.54, 21.10)	
*p* value	0.300	
CFB in TSAT (%) to Week 36		
N	212	203
Baseline, mean (SD)	33.58 (13.77)	34.62 (12.39)
Week 36 n	151	148
CFB, mean (SD)	−2.22 (18.44)	−2.79 (15.93)
LS mean (95% CI)	−4.29 (−9.16, 0.58)	−2.60 (−7.94, 2.74)
LSMD ^c^ (95% CI)	−1.69 (−6.90, 3.52)	
*p* value	0.524	
CFB in ferritin (ng/mL) to Week 36		
N	212	203
Baseline, mean (SD)	488.76 (415.64)	531.69 (393.06)
Week 36 n	152	152
CFB, mean (SD)	−139.06 (327.04)	−79.85 (260.40)
LS mean (95% CI)	−101.70 (−191.04, −12.35)	−52.44 (−150.64, 45.76)
LSMD ^d^ (95% CI)	−49.25 (−145.36, 46.86)	
*p* value	0.314	

ANCOVA, analysis of covariance; CFB, change from baseline; ESA, erythropoiesis-stimulating agent; Hb, hemoglobin; LS, least squares; LSMD, least squares mean difference; SD, standard deviation; TSAT, transferrin saturation. ^a^ Treatment comparison was made using an ANCOVA model with baseline Hb and baseline hepcidin as covariates, and study, treatment, study-by-treatment interaction, history of cardiovascular/cerebrovascular/thromboembolic diseases (yes vs. no), and mean prescribed baseline epoetin alfa dose or equivalent (≤150 vs. >150 IU/kg/week) as fixed effects. ^b^ Treatment comparison was made using an ANCOVA model with baseline Hb and baseline iron as covariates, and study, treatment, study-by-treatment interaction, history of cardiovascular/cerebrovascular/thromboembolic diseases (yes vs. no), and mean prescribed baseline epoetin alfa dose or equivalent (≤150 vs. >150 IU/kg/week) as fixed effects. ^c^ Treatment comparison was made using an ANCOVA model with baseline Hb and baseline TSAT as covariates, and study, treatment, study-by-treatment interaction, history of cardiovascular/cerebrovascular/thromboembolic diseases (yes vs. no), and mean prescribed baseline epoetin alfa dose or equivalent (≤150 vs. >150 IU/kg/week) as fixed effects. ^d^ Treatment comparison was made using an ANCOVA model with baseline Hb and baseline ferritin as covariates, and study, treatment, study-by-treatment interaction, history of cardiovascular/cerebrovascular/thromboembolic diseases (yes vs. no), and mean prescribed baseline epoetin alfa dose or equivalent (≤150 vs. >150 IU/kg/week) as fixed effects.

**Table 4:** Treatment-emergent adverse events (OT-28).

	n (%), IR/100 PEY	
	Roxadustat (N = 215) PEY = 336.2	ESA (N = 204) PEY = 323.7
TEAE	190 (88.4), 56.5	176 (86.3), 54.4
Serious TEAE	125 (58.1), 37.2	116 (56.9), 35.8
TEAE leading to discontinuation of study drug	19 (8.8), 5.7	11 (5.4), 3.4
Grade ≥ 3 TEAE	93 (43.3), 27.7	89 (43.6), 27.5
TEAE leading to death	27 (12.6), 8.0	26 (12.7), 8.0

PEY for each patient = ([last dose date − first dose date] + 1)/365.25. ESA, erythropoiesis-stimulating agent; IR, incidence rate; IR/100 PEY = 100 × number of patients with events/PEY; OT-28, on-treatment period plus 28 days; PEY, patient exposure years; TEAE, treatment-emergent adverse event.

**Table 5:** Summary of MACE, MACE+, and ACM safety endpoints (OT-7) for patients on peritoneal dialysis.

	Roxadustat (N = 215) PEY = 336.2	ESA (N = 204) PEY = 323.7
**MACE**		
Events, n (%)	27 (12.6)	29 (14.2)
IR/100 PEY	8.0	9.0
HR (95% CI) ^a^	0.96 (0.54, 1.71)	
**MACE+**		
Events, n (%)	30 (14.0)	38 (18.6)
IR/100 PEY	8.9	11.7
HR (95% CI) ^a^	0.89 (0.53, 1.50)	
**ACM**		
Events, n (%)	19 (8.8)	20 (9.8)
IR/100 PEY	5.7	6.2
HR (95% CI) ^a^	0.96 (0.48, 1.92)	

PEY for each patient = (last dose date − first dose date + 1)/365.25. ACM, all-cause mortality; CI, confidence interval; ESA, erythropoiesis-stimulating agent; HR, hazard ratio; IR, incidence rate; IR/100 PEY = 100 × number of patients with events/PEY; MACE, major adverse cardiovascular event; MACE+, MACE plus congestive heart failure or unstable angina requiring hospitalization; OT-7, on-treatment period plus 7 days; PEY, patient exposure years. ^a^ Hazard ratios comparing roxadustat to ESA were derived using a meta-analysis method, which combined the individual study log–hazard ratios with the weight inversely proportional to the variance of the study-specific log–hazard ratios.

**Table 6:** Components of MACE and MACE+ (OT-7) for patients on peritoneal dialysis.

	n (%), FAIR/100 PY	
	Roxadustat (N = 215)	ESA (N = 204)
Components of MACE	27 (12.6), 8.1	29 (14.2), 9.0
ACM	17 (7.9), 5.1	16 (7.8), 5.0
MI	6 (2.8), 1.8	8 (3.9), 2.5
Stroke	4 (1.9), 1.2	5 (2.5), 1.6
Components of MACE+	30 (14.0), 9.1	38 (18.6), 12.1
ACM	15 (7.0), 4.5	15 (7.4), 4.8
MI	6 (2.8), 1.8	8 (3.9), 2.5
Stroke	4 (1.9), 1.2	5 (2.5), 1.6
Unstable angina requiring hospitalization	1 (0.5), 0.3	0 (0.0), 0.0
Hospitalization for CHF	4 (1.9), 1.2	10 (4.9), 3.2

PY for each patient = ([first event occurrence or censor date − first dose date] + 1)/365.25. Components of MACE PY = (roxadustat: 333.4; ESA: 322.6). Components of MACE+ PY = (roxadustat: 329.8; ESA: 315.3). ACM, all-cause mortality; CHF, congestive heart failure; ESA, erythropoiesis-stimulating agent; FAIR, follow-up adjusted incidence rate; FAIR/100 PY = 100 × number of patients with events/PY; MACE, major adverse cardiovascular event (MI, stroke, or ACM; the first event of the 3 is counted in this table); MACE+, a composite of MACE plus unstable angina or CHF requiring hospitalization up to 7 days after the last dose; MI, myocardial infarction; OT-7, on-treatment period plus 7 days; PY, patient years.

**Table 7:** Primary cause of mortality (OT-7) for patients on peritoneal dialysis.

	N (%), IR/100 PEY	
	Roxadustat (N = 215) PEY = 336.2	ESA (N = 204) PEY = 323.7
Total mortality	19 (8.8), 5.7	20 (9.8), 6.2
CV-related	8 (3.7), 2.4	11 (5.4), 3.4
Acute MI	2 (0.9), 0.6	1 (0.5), 0.3
Sudden cardiac death	4 (1.9), 1.2	5 (2.5), 1.5
Heart failure	1 (0.5), 0.3	1 (0.5), 0.3
CV hemorrhage	0 (0.0), 0.0	2 (1.0), 0.6
Stroke	0 (0.0), 0.0	2 (1.0), 0.6
Other CV causes	1 (0.5), 0.3	0 (0.0), 0.0
Non-CV-related	7 (3.3), 2.1	8 (3.9), 2.5
Pulmonary	1 (0.5), 0.3	0 (0.0), 0.0
Renal	0 (0.0), 0.0	2 (1.0), 0.6
Infection	3 (1.4), 0.9	6 (2.9), 1.9
Hemorrhage	1 (0.5), 0.3	0 (0.0), 0.0
Inflammatory immune/autoimmune	1 (0.5), 0.3	0 (0.0), 0.0
Neurological	1 (0.5), 0.3	0 (0.0), 0.0
Undetermined	4 (1.9), 1.2	1 (0.5), 0.3

PEY for each patient = ([last dose date − first dose date] + 1)/365.25. CV, cardiovascular; ESA, erythropoiesis-stimulating agent; IR, incidence rate; IR/100 PEY = 100 × number of patients with events/PEY; MI, myocardial infarction; OT-7, on-treatment period plus 7 days; PEY, patient exposure years.

## Data Availability

Researchers may request access to anonymized participant-level data, trial-level data, and protocols from Astellas-sponsored clinical trials at www.clinicalstudydatarequest.com. For the Astellas criteria on data sharing, see https://clinicalstudydatarequest.com/Study-Sponsors/Study-Sponsors-Astellas.aspx (Accessed on 5 September 2024). The datasets generated during and/or analyzed during the current study are available from the corresponding author upon reasonable request.

## Supplementary Materials

The following supporting information can be downloaded at https://www.mdpi.com/article/10.3390/jcm13226729/s1, Abbreviations; Plain Language Summary; Supplementary Methods; Figure S1: Mean Hb levels (g/dL, lines) and mean weekly total dose (columns) of roxadustat (A) or ESA (B) from baseline to Week 104 in patients with baseline C-reactive protein level above the upper limit of normal; Table S1: Baseline covariates for endpoints/parameters; Table S2: Duration of treatment exposure; Table S3: Treatment-emergent adverse events occurring in ≥5% of patients in either treatment group (OT-28).
