# Supplementary material for: Roxadustat Efficacy and Safety in Patients Receiving Peritoneal Dialysis: Pooled Analysis of Four Phase 3 Studies
Source: J Clin Med. 2024 Nov 8;13(22):6729. doi: 10.3390/jcm13226729 (PMC11595076; doi:10.3390/jcm13226729)
Supplement: Supplementary file 1 [file jcm-13-06729-s001.zip › jcm-3225913-supplementary.pdf]

Supplementary Materials

Abbreviations

Lay Summary

Supplementary Methods

Table S1

Table S2

Table S3

Figure S1

Abbreviations

ACM, all-cause mortality

ANCOVA, analysis of covariance

CFB, change from baseline

CHF, congestive heart failure

CI, confidence interval

CKD, chronic kidney disease

CV, cardiovascular

DA, darbepoetin alfa

DD, dialysis-dependent

EPO-α, epoetin alfa

ESA, erythropoiesis-stimulating agent

FAIR, follow-up adjusted incidence rate

Hb, hemoglobin

HD, hemodialysis

HIF-PH, hypoxia-inducible factor prolyl hydroxylase

HR, hazard ratio

hsCRP, high-sensitivity C-reactive protein

IR, incidence rate

LDL-C, low-density lipoprotein cholesterol

LS, least squares

LSMD, least squares mean difference

MACE, major adverse cardiovascular event

MACE+, major adverse cardiovascular event plus unstable angina or congestive heart failure

MI, myocardial infarction

NA, not applicable

OT-7, on-treatment period plus 7 days

OT-28, on-treatment period plus 28 days

PD, peritoneal dialysis

PEY, patient exposure years

PY, patient years

QoL, quality of life

RBC, red blood cell

SD, standard deviation

SEM, standard error of the mean

TE, thromboembolic

TEAE, treatment-emergent adverse event

TSAT, transferrin saturation

ULN, upper limit of normal

LAY SUMMARY

Roxadustat is a tablet medicine that treats anemia, or a lower-than-normal amount of healthy red blood cells, in patients with chronic kidney disease. Anemia severity is assessed by measuring levels of hemoglobin, the protein that carries oxygen within red blood cells. This study used data from four clinical trials to examine how effective and safe roxadustat was compared with the current standard of care (injected erythropoiesis-stimulating agents) in patients with anemia of chronic kidney disease receiving peritoneal dialysis, a form of at-home dialysis. Roxadustat was as effective as erythropoiesis-stimulating agents in raising hemoglobin levels. Fewer patients treated with roxadustat required a blood transfusion compared with patients treated with erythropoiesis-stimulating agents. The safety of roxadustat was comparable to that of erythropoiesis-stimulating agents. Roxadustat, a medication with a novel mechanism of action, is an alternative for treating anemia of chronic kidney disease in patients receiving peritoneal dialysis.

Supplementary Methods

Rescue Therapy

Patients treated with roxadustat were permitted to use ESAs if: after the maximum dose of roxadustat or two consecutive dose increases, based on clinician opinion, hemoglobin concentration had not adequately responded; another cause for lack of hemoglobin response was not clinically determined; transplant-eligible patients wanted to reduce the risk of alloimmunization; or hemoglobin concentration was <8.5 g/dL (SIERRAS and ROCKIES) or <9.0 g/dL (PYRENEES) following two consecutive measurements ≥5 days apart.

Iron Administration Protocols

All studies permitted oral iron supplementation. In the ROCKIES and PYRENEES studies, roxadustat-treated patients could be given intravenous iron if, after ≥2 dose increases, hemoglobin levels did not increase sufficiently, and transferrin saturation (TSAT) or ferritin levels were <20% or <100 ng/mL, respectively. In the HIMALAYAS and SIERRAS studies, uniform intravenous iron supplementation was permitted in both treatment arms if, in the investigator's opinion, hemoglobin levels did not respond adequately and TSAT or ferritin levels were <20% or <100 ng/mL, respectively [1].

Statistical Analyses

The analysis for hemoglobin change from baseline (CFB) used a mixed model of repeated measures with baseline hemoglobin as a continuous covariate, and study,

treatment, visit, visit-by-treatment interaction, study-by-treatment interaction, and history of cardiovascular/cerebrovascular/thromboembolic diseases (yes vs no) as fixed effects. For hemoglobin CFB for Weeks 28–52 regardless of rescue therapy, and for hemoglobin CFB for Weeks 18–24 regardless of rescue therapy limited to those patients with baseline high-sensitivity C-reactive protein levels above the upper limit of normal, the treatment comparison between roxadustat and erythropoiesis-stimulating agents was made using a multiple imputation strategy by combining the results of an analysis of covariance (ANCOVA) model with baseline hemoglobin as the covariate and study, treatment, study-by-treatment interaction, and history of cardiovascular/cerebrovascular/thromboembolic diseases (yes vs no) as fixed effects.

For low-density lipoprotein cholesterol (LDL-C) CFB to Weeks 12–28, treatment comparison was made using an ANCOVA model with baseline hemoglobin and baseline LDL-C as covariates, and study, treatment, study-by-treatment interaction, and history of cardiovascular/cerebrovascular/thromboembolic diseases (yes vs no) as fixed effects. For iron parameters CFB for Week 24 (hepcidin) or Week 36 (serum iron, TSAT, ferritin), treatment comparisons were made using ANCOVA models with baseline hemoglobin and baseline [iron parameter] as covariates, and study, treatment, study-by-treatment interaction, history of cardiovascular/cerebrovascular/thromboembolic diseases (yes vs no), and mean prescribed baseline epoetin alfa dose or equivalent (≤150 vs >150 IU/kg/week) as fixed effects.

REFERENCES

20. Pergola, P.E.; Charytan, C.; Little, D.J.; Tham, S.; Szczech, L.; Leong, R.; Fishbane, S. Changes in iron availability with roxadustat in nondialysis- and dialysis-dependent patients with anemia of CKD. *Kidney360* **2022**, *3*, 1511-1528, doi: 10.34067/kid.0001442022.

Table S1: Baseline covariates for endpoints/parameters

Endpoint/Parameter	Baseline Covariates
**CFB in Hb (g/dL) to Weeks 28–36, without rescue therapy**	Hemoglobin
**CFB in Hb (g/dL) to Weeks 28–52 regardless of rescue therapy**	Hemoglobin
**CFB in Hb (g/dL) to Weeks 18–24, regardless of rescue therapy, for patients with baseline hsCRP >ULN**	Hemoglobin
**CFB in LDL-C (mg/dL) to Weeks 12–28**	Hemoglobin<br>LDL-C
**CFB in serum hepcidin (µg/L) to Week 24**	Hemoglobin<br>Serum hepcidin
**CFB in serum iron (µg/dL) to Week 36**	Hemoglobin<br>Serum iron
**CFB in TSAT (%) to Week 36**	Hemoglobin<br>TSAT
**CFB in ferritin (ng/mL) to Week 36**	Hemoglobin<br>Ferritin

Table S2: Duration of treatment exposure.

Parameter	Roxadustat (N=215)	ESA (N=207)
Duration of exposure (weeks), mean (SD)	81.6 (55.0)	82.8 (55.7)
Mean exposure (years)	1.56	1.59
PEY	336.2	323.7

ESA, erythropoiesis-stimulating agent; PEY, patient exposure years; SD, standard deviation.

Table S3: Treatment-emergent adverse events occurring in ≥5% of patients in either treatment group (OT-28).

	N (%), Incidence Rate/100 PEY	
	**Roxadustat (N=215) PEY=336.2**	**ESA (N=204) PEY=323.7**
Gastrointestinal disorders	49 (22.8), 14.6)	46 (22.5), 14.2
Abdominal pain	11 (5.1), 3.3	9 (4.4), 2.8
Constipation	14 (6.5), 4.2	10 (4.9), 3.1
Diarrhea	19 (8.8), 5.7	17 (8.3), 5.3
Nausea	18 (8.4), 5.4	21 (10.3), 6.5
Vomiting	12 (5.6), 3.6	17 (8.3), 5.3
General disorders and administration-site conditions	33 (15.3), 9.8	24 (11.8), 7.4
Fatigue	10 (4.7), 3.0	13 (6.4), 4.0
Peripheral edema	13 (6.0), 3.9	6 (2.9), 1.9
Pyrexia	13 (6.0), 3.9	10 (4.9), 3.1
Infections and infestations	95 (44.2), 28.3	90 (44.1), 27.8
Catheter-site infection	8 (3.7), 2.4	13 (6.4), 4.0
Peritonitis	59 (27.4), 17.5	59 (28.9), 18.2
Pneumonia	15 (7.0), 4.5	12 (5.9), 3.7
Upper respiratory tract infection	11 (5.1), 3.3	9 (4.4), 2.8
Viral upper respiratory tract infection	15 (7.0), 4.5	11 (5.4), 3.4
Metabolism and nutrition disorders	25 (11.6), 7.4	21 (10.3), 6.5
Hyperkalemia	12 (5.6), 3.6	6 (2.9), 1.9
Hypokalemia	7 (3.3), 2.1	11 (5.4), 3.4
Nervous system disorders	28 (13.0), 8.3	17 (8.3), 5.3
Dizziness	11 (5.1), 3.3	6 (2.9), 1.9
Headache	17 (7.9), 5.1	12 (5.9), 3.7
Psychiatric disorders	11 (5.1), 3.3	6 (2.9), 1.9
Insomnia	11 (5.1), 3.3	6 (2.9), 1.9
Respiratory, thoracic, and mediastinal disorders	13 (6.0), 3.9	23 (11.3), 7.1
Cough	5 (2.3), 1.5	15 (7.4), 4.6
Dyspnea	10 (4.7), 3.0	13 (6.4), 4.0
Vascular disorders	54 (25.1), 16.1	50 (24.5), 15.4
Hypertension	28 (13.0), 8.3	25 (12.3), 7.7
Hypertensive crisis	14 (6.5), 4.2	12 (5.9), 3.7
Hypotension	20 (9.3), 5.9	17 (8.3), 5.3

n = number of participants with the specified event.

Subjects with more than one event in a category are counted only once for that category.
PEY for each patient = ([last dose date − first dose date] + 1) / 365.25.
Incidence rate/100 PY = 100 x number of patients with events/PY.

ESA, erythropoiesis-stimulating agent; OT-28, on-treatment period plus 28 days; PEY, patient exposure years.

Figure S1: Mean Hb levels (g/dL, lines) and mean weekly total dose (columns) of roxadustat (A) or ESA (B) from baseline to Week 104 in patients with baseline C-reactive protein level above the upper limit of normal. ESA, erythropoiesis-stimulating agent; Hb, hemoglobin; SE, standard error.

(A)

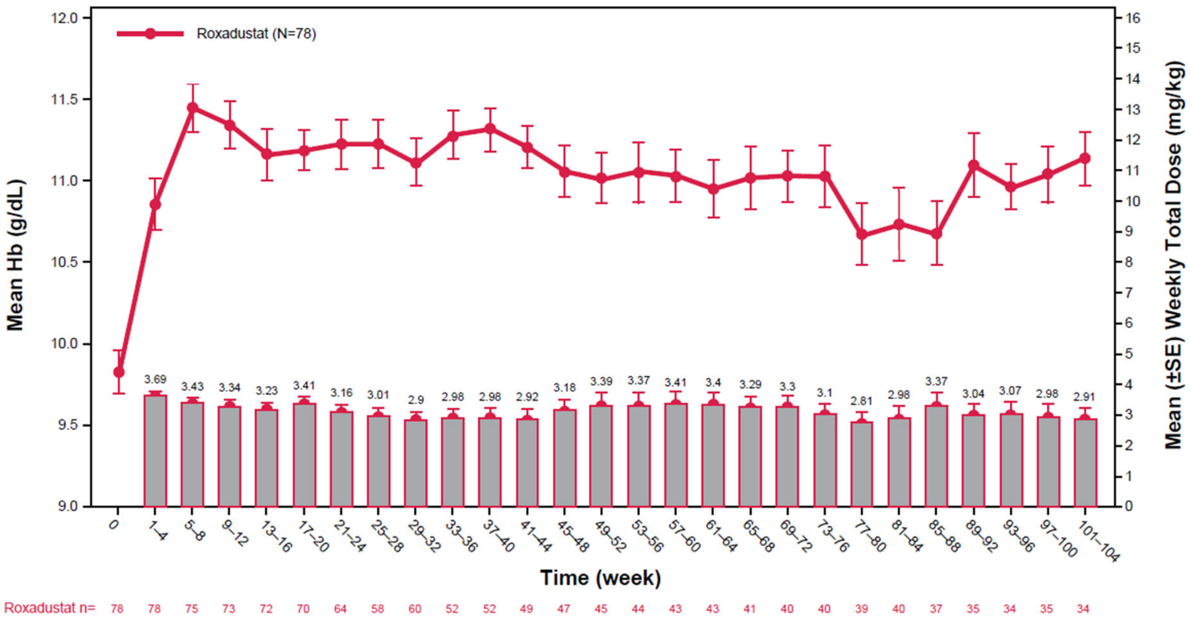

(B)

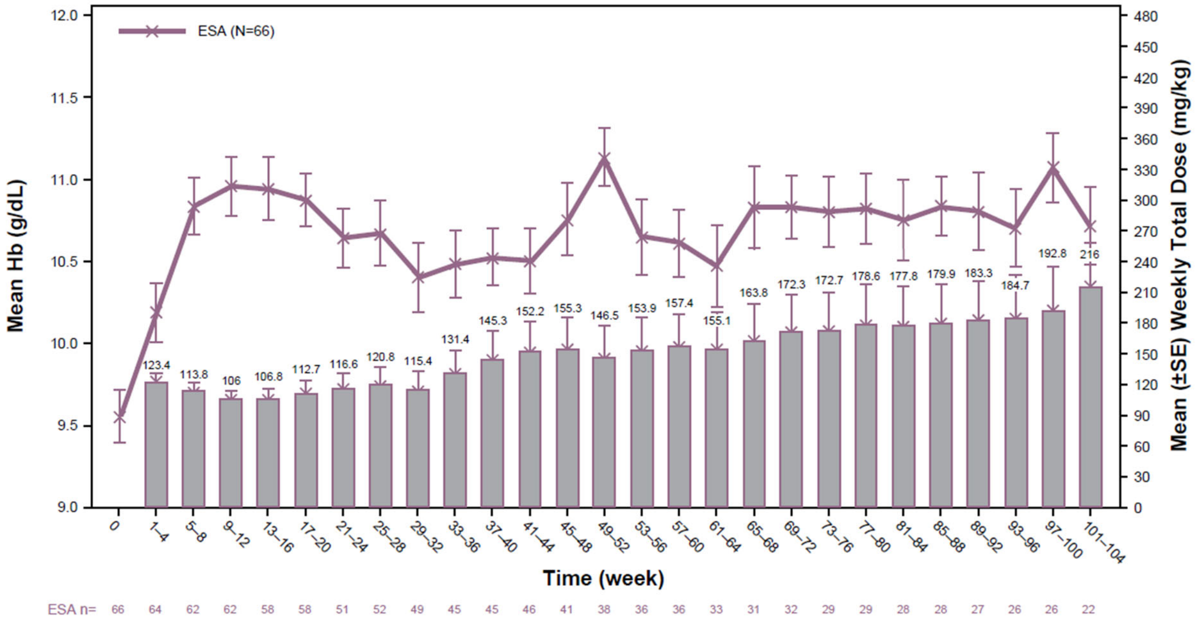

ESA (N=66)
Mean Hb (g/dL)
Mean (±SE) Weekly Total Dose (mg/kg)
Time (week)
ESA n= 66 64 62 62 58 58 51 52 49 45 45 46 41 38 36 36 33 31 32 29 29 28 28 27 26 26 22
123.4 113.8 106 106.8 112.7 116.6 120.8 115.4 131.4 145.3 152.2 155.3 146.5 153.9 157.4 155.1 163.8 172.3 172.7 178.6 177.8 179.9 183.3 184.7 192.8 216
